# Turcot Syndrome: A Synchronous Clinical Presentation of Glioblastoma Multiforme and Adenocarcinoma of the Colon

**DOI:** 10.1155/2012/720273

**Published:** 2012-10-16

**Authors:** Sabiq Dipro, Faisal Al-Otaibi, Adel Alzahrani, Anwar Ulhaq, Essam Al Shail

**Affiliations:** ^1^Alfaisal University, Riyadh 11533, Saudi Arabia; ^2^Division of Neurological Surgery, Department of Neurosciences, King Faisal Specialist Hospital & Research Center, P.O. Box 3354, Riyadh 11211, Saudi Arabia

## Abstract

Turcot syndrome (TS) is a rare hereditary disorder clinically characterized by the occurrence of primary tumors of the colon and the central nervous system (CNS). Here we present the case of an 11-year-old boy with a synchronous clinical presentation of both glioblastoma multiforme (GBM) and colonic adenocarcinoma. A molecular genetic study revealed microsatellite instability in the DNA mismatch repair (MMR) gene. This patient ultimately survived for 13 months after clinical presentation. Based on this case study, the synchronous presentation of glioblastoma multiforme and adenocarcinoma of the colon might suggest a shorter survival rate for patients with Turcot syndrome. A literature review complements this paper.

## 1. Introduction

Turcot syndrome (TS) was originally described by Turcot in 1959 [1–3]. It is characterized by the development of primary tumors of the central nervous system (CNS), such as glioblastoma multiforme (GBM) and medulloblastoma, along with numerous adenomatous colorectal polyps and colonic adenocarcinoma. TS usually develops in the patient's teens and can be genetically distinguished as either familial adenomatous polyposis (FAP) or hereditary nonpolyposis colon carcinoma (HNPCC) [4]. The syndrome was divided into two types by Paraf and colleagues in 1997 [5]. TS type I is characterized by the presence of glial tumors, relatively few colonic polyps, and cancer. TS type II is characterized by thousands of colonic polyps and increased risk of medulloblastoma.

Since the time when this syndrome was first identified, there have only been isolated case reports or case series with a limited number of patients being discussed in the literature. The synchronous clinical presentation of CNS tumors and colonic adenocarcinoma in this syndrome is extremely rare. In this paper, we present a case of TS with simultaneous clinical presentation of GBM and colonic adenocarcinoma.

## 2. Case Report

An 11-year-old boy presented with headache for the past 5 days, vomiting, and history of a single tonic clonic seizure. He had no history of weakness, loss of consciousness, or memory disturbance. He was also suffering from vague abdominal pain and loose motion over a period of one month prior to presentation. He had positive family history of colonic cancer. His sister had multiple café-au-lait spots and multiple colonic polyps.

**Figure 1:**
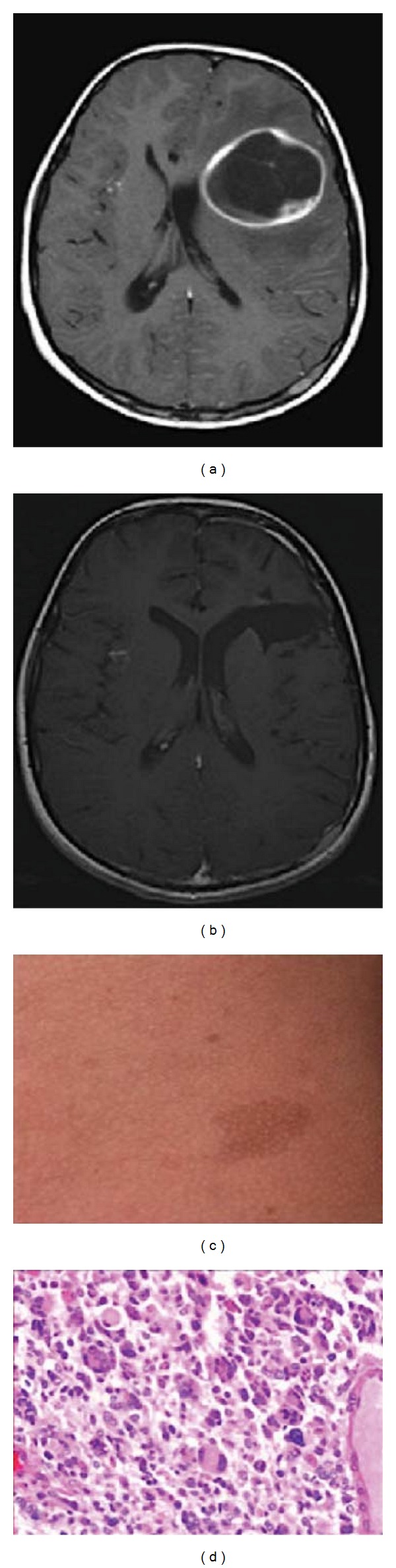
T1 axial MRI brain depicting left frontal tumor with rim enhancement associated with midline shift, (a) and after resection MRI brain demonstrates no residual tumor and a resolved mass effect (b). Photograph of the patient skin café-au-lait spots (c). (d) Histopathology shows high mitotic index and multinucleated giant cells representing GBM features. Hematoxylin and eosin stain 5 400.

During clinical examination, he was fully awake and oriented with a Glasgow coma scale (GCS) score of 15. His pupils were bilaterally equal and reacting; no motor or sensory deficit was noticed. His gait and posture were normal. On his skin there were multiple café-au-lait spots and areas of hypopigmentation (Figure 1). Brain CT showed that he had a 4 × 4 cm left frontal cystic tumor with perifocal edema, effacement of left lateral ventricle, and subfalcine herniation. The brain appeared tight. The tumor had an intense ring-enhancing rim. He underwent left-frontal craniotomy and gross total resection of the tumor. Histopathology showed GBM with giant-cell features (WHO grade IV) (Figure 1). Immunohistochemistry of the tumor showed positive p53 and Ki-67 >90%.

**Figure 2:**
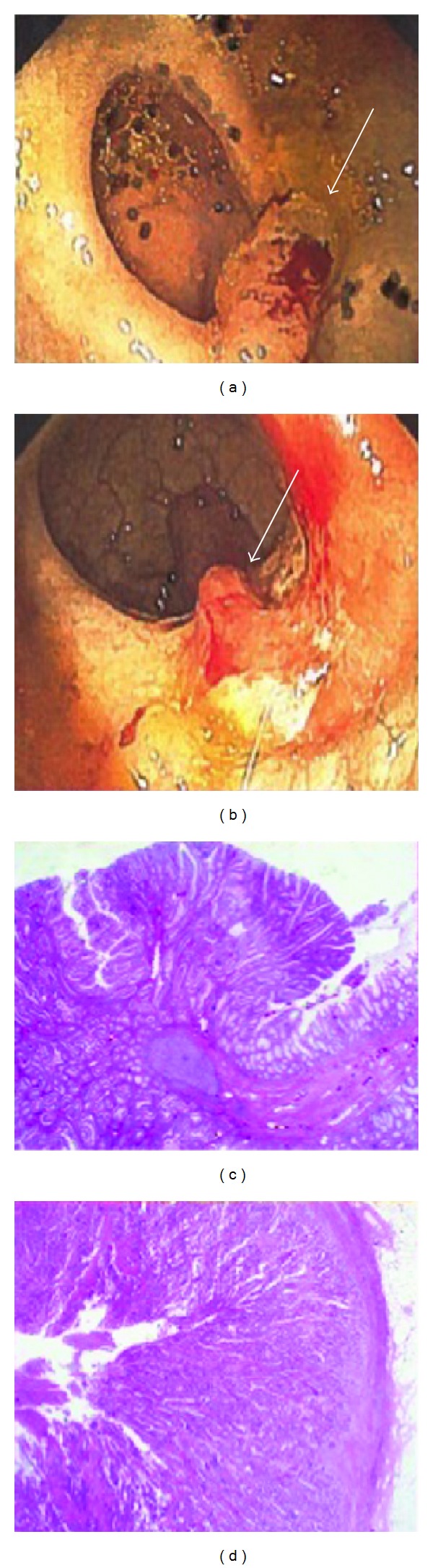
Colon fiberscopy demonstrates ulcerative adenoma (arrow; (a), (b)). Histopathology slides reveal the adenoma, hematoxylin and eosin stain 5 100. (c) and carcinoma invasion of the colon muscular layer, hematoxylin and eosin stain 5 200 (d).

He continued to have abdominal pain and diarrhea and developed melena and intermittent frank bleeding from the rectum. His blood cell count, platelets, and renal and coagulation profiles were within the normal range. Upper GI endoscopy was normal; however, colonoscopy showed multiple colonic polyps of various sizes with ulceration and active bleeding (Figure 2). Punch biopsy of the polyps revealed grade 2 adenocarcinoma (Figure 2). A molecular genetic workup of the patient showed mutation in the DNA mismatch repair (MMR) gene with microsatellite instability. He was diagnosed with Turcot syndrome with cerebral GBM and adenocarcinoma of the colon. For adenocarcinoma of the colon, he underwent laparoscopic total colectomy and ileocolic anastomosis. A month later, he had recurrent bleeding from the rectum and developed new rectal polyps. To address this issue, he underwent proctectomy and terminal ileostomy. The patient received adjuvant radiotherapy for the brain cancer and chemoradiotherapy for the colonic cancer.

**Figure 3:**
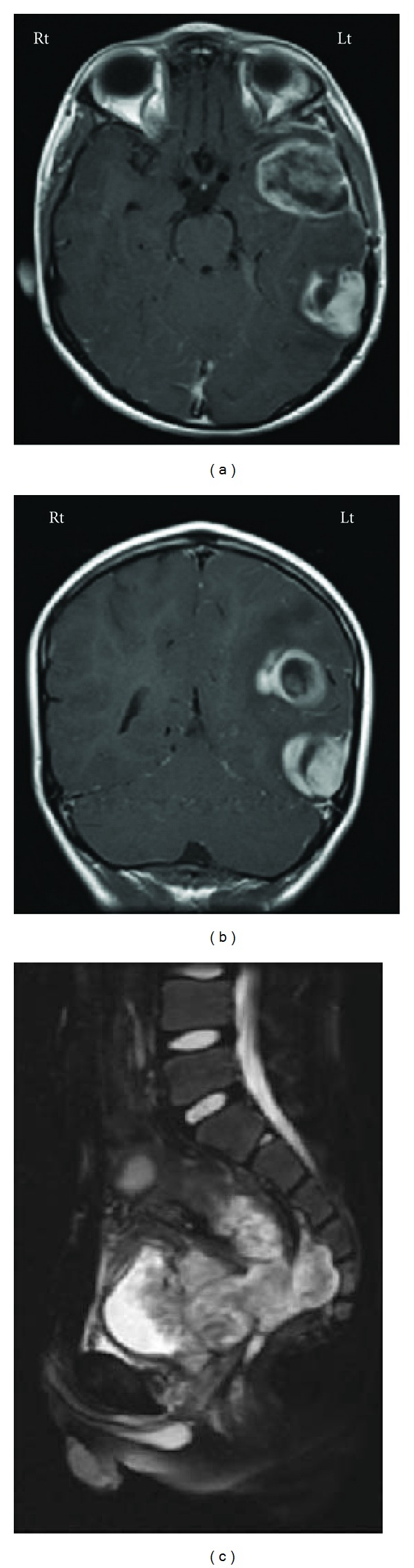
MRI brain shows the dissemination of the GBM in the left hemisphere ((a) (b)). (c) MRI pelvis (sagittal) demonstrates extensive colorectal adenocarcinoma recurrence.

Nine months later, he presented with headache and was found to have mild right hemiparesis with an overall Karnofsky performance score of over 90. CT and MRI of the brain showed a left temporal ring-enhancing lesion about 4 × 5 cm in size with uncal herniation (Figure 3). He also had another lesion at the site of the previous surgery. He underwent redo left temporal craniotomy and gross total resection of the tumor. The biopsy report revealed pathological features similar to those of GBM. He was started on temozolomide as palliative therapy. Three months later, he had multiple recurrences and dissemination of the cerebral tumors on the left temporal, multifocal frontal, and parietal areas. At the same time, he had a massive progression of colonic carcinoma (Figure 3). He died 13 months after the initial diagnosis. 

## 3. Discussion

Turcot syndrome is also known as brain-tumor polyposis and is characterized by the occurrence of primary tumors of the CNS and multiple colorectal adenomas and/or colonic adenocarcinoma [5, 11]. From a genetic standpoint, TS can be divided into two subtypes: the first is manifested by the MMR gene and PMS2 with a high risk of developing GBM, and the second type is manifested by the adenomatous polyposis coli gene germline mutation with a high risk of developing medulloblastoma [5, 16]. However, this classification cannot be applied to all cases reported in the literature. There are reports of cases with the occurrence of both GBM and medulloblastoma, as in the report from McLaughlin and colleagues [12]. Nevertheless, their patient was treated for medulloblastoma and received whole-brain radiation therapy 10 years prior to the diagnosis of GBM. Therefore, radiation-induced GBM cannot be excluded. Moreover, the occurrence of tumors other than GBM and medulloblastoma has been reported [17]. Based on TS classification, our patient fit the clinical and molecular genetic features of TS type I. 

The pathological differentiation between sporadic GBM and TS-associated GBM has not been adequately addressed in the literature. Recently, Lusis and colleagues reported on three cases involving patients with TS-associated GBM [11]. The histopathological and molecular genetic features in all three cases consisted of giant-cell GBM, and the patients in two of the cases had sarcomatous changes. The Ki-67 indices were found to range between 18% and 45%, and all three GBMs demonstrated positive nuclear p53. The first two patients survived 44 and 55 months, respectively, and the last patient survived 29 months and was still alive when the report was published. For our patient, the Ki-67 was >90% and the tumor showed p53 positivity in addition to MMR gene mutation. The tumor did exhibit giant-cell features. The patient survived only 13 months after initial clinical presentation. The average survival rate of patients with TS and GBM was found to be 27 months; however, when the investigators excluded the two patients with the longest-term survival, the average survival rate dropped to 16 months [18, 19]. In contrast, the survival rate of sporadic GBM has been found to be around 15 months [20]. GBM and anaplastic astrocytoma are the most commonly reported CNS tumor associated with TS. In a review of 33 reported cases, 21 reports were for GBM and anaplastic astrocytoma [21].

Most of the reported TS cases in the literature are characterized by different time intervals between the clinical presentation of CNS tumors and the colonic adenocarcinoma [18, 22]. The presence of symptomatic CNS GBM and colorectal cancers at the same time carries more management difficulties and, more important, might shorten the survival rate. Our patient presented with CNS and gastrointestinal symptoms simultaneously. We elected to treat the CNS tumor first due to the presence of mass effects that were causing increased intracranial pressure. He was operated on for the GBM recurrence; however, a few months later he had a disseminated GBM involving almost the entire left cerebrum that was not amenable to further surgical intervention. This indicates highly malignant disease. The presence of GBM in TS may suggest a lower survival rate in contrast to other CNS tumors in most reported cases.  Table 1 summarizes selected studies demonstrating the survival rates of TS after the time of high-grade glioma diagnosis.

## 4. Conclusion

Simultaneous clinical presentation of CNS GBM and adenocarcinoma of the colon in TS might be an indicator of a shorter survival rate. Moreover, the presence of GBM may also negatively affect the survival rate in contrast to other types of TS-associated CNS tumors.

## Figures and Tables

**Table 1:** Summary of selected reported cases with high-grade glioma and adenocarcinoma of the colon.

Authors (year)	No. of cases	CNS tumors	Other associated tumors	Interval between the diagnosis of high-grade glioma and colonic adenocarcinoma	Survival rate after the diagnosis of high-grade glioma
Agostini et al. (2005) [6]	1	Right frontal giant-cell GBM	(i) Adenocarcinoma of the ampullary region (ii) Adenomas of the transverse and sigmoid colon	9 months	Not reported
Castillo and Wilson (2002) [7]	1	Left frontoparietal GBM	Multiple colonic polyps with adenocarcinoma	Same time as diagnosis	Not reported
Chung et al. (2012) [8]	1	Right parietotemporal GBM	(i) Ovarian cystadenocarcinoma (ii) Adenocarcinoma in sigmoid colon	12 years	Still alive at the age of 25 at the time of reporting the case
Eguchi et al. (1993) [9]	1	Recurring grade 3 astrocytoma in the right parietal lobe	Adenocarcinoma of the colon	Same time as diagnosis	One year
Kleinerman et al. (2012) [10]	1	Malignant astrocytoma (grade III) of the frontal lobe	(i) Recurring colon cancer (ii) Carcinoma of the right groin (iii) Nodular basal cell of the right nose and the left cheek	12 years	One year
Lusis et al. (2010) [11]	3	GBM	Colorectal adenocarcinoma	3, 6, and 6 months, respectively	Two of the patients died after 44 and 55 months of diagnosis, and the third was still alive at the time of writing the article. Survived >29 months since diagnosis
McLaughlin et al. (1998) [12]	1	(i) Medulloblastoma (ii) Right parietal GBM	Adenocarcinoma of the colon	About 10 years	A few months
Radin et al. (1984) [13]	1	GBM of the cervical spinal cord	Adenocarcinoma of the descending colon	4 months	6 months
Schroder et al. (1983) [14]	1	GBM of the left frontal lobe	Carcinoma of the jejunum	5 years	The patient died from brain herniation due to GBM acute presentation. The diagnosis was made from autopsy
Takayama et al. (1989) [15]	1	Initial diagnosis was grade I astrocytoma of left frontal lobe, and the recurrence was GBM	Adenocarcinoma of the ascending colon	6 months	18 months
